# What motivated residents of Saudi Arabia to receive the COVID-19 vaccine?

**DOI:** 10.3389/fpubh.2023.1065157

**Published:** 2023-02-07

**Authors:** Jenny Gray, Ahmed AlHumaidi AlAnazi, Fahad AlSumait, Amani Abu-Shaheen, Muhammad Salman Bashir, Mohammed Al Sheef

**Affiliations:** ^1^Dentistry Administration, King Fahad Medical City, Riyadh Second Health Cluster, Riyadh, Saudi Arabia; ^2^Department of Medicine, King Fahad Medical City, Riyadh Second Health Cluster, Riyadh, Saudi Arabia; ^3^Research Center, King Fahad Medical City, Riyadh Second Health Cluster, Riyadh, Saudi Arabia; ^4^Department of Biostatistics, Research Center, King Fahad Medical City, Riyadh Second Health Cluster, Riyadh, Saudi Arabia

**Keywords:** Saudi Arabia, COVID-19, vaccine, SARS CoV-2, population

## Abstract

**Background:**

Acceptance of vaccination is a multifactorial issue. The unprecedented speed at which the COVID-19 disease spread globally has meant that people have had to face the idea of receiving novel vaccines for a novel disease.

**Purpose:**

Studies conducted earlier in the pandemic had shown high vaccine hesitancy in Saudi Arabia, therefore we wanted to understand the motivating factors for people living in Saudi Arabia with regards to accepting the COVID-19 vaccine, our survey was conducted when the government had already mandated vaccination to enter public spaces. Saudi society is not particularly outspoken and therefore it was of special importance to the authors to explore the motivation behind COVID-19 vaccines.

**Methods:**

This is a cross-sectional survey of 802 participants living in Saudi Arabia. The questionnaire was distributed to staff, visitors, and patients in a hospital in Saudi Arabia and *via* electronic means to the general population.

**Results:**

A total of 521 (65%) of the respondents were women, and 281 (35%) were men. A total of 710 (88.5%) were Saudi, and 55 (6.9%) were non-Saudi. The majority of participants (496, 65.7%) stated that they registered for the vaccine as soon as it was available, with 185 (24.5%) stating that they registered when they were mandated to do so and 74 (9.8%) registered only when they felt cases were increasing. Most participants (316, 41%) stated that the main reason for taking the vaccine was one of a self-protective nature, followed by indirect vaccination (240, 31.1%), paternalistic reasons (157, 20.4%) and altruistic reasons (58, 7.5%).

**Conclusions:**

With the increased burden on healthcare that is being faced by COVID-19, other resources need to be carefully allocated. This paper may aid the Saudi government in understanding the motivation for the population to take the vaccine and therefore facilitate any future vaccination campaigns to ensure the best utilization of resources.

## Introduction

In December 2019, a novel virus was discovered, which was later named “severe acute respiratory syndrome coronavirus 2” (SARS CoV-2); later, the disease was more commonly known as COVID-19. The associated virus has had a devastating global impact.

Vaccine research began as soon as it became evident that self-limiting measures, such as social distancing and lockdowns, were not a practical long-term solution and that a pharmaceutical intervention would be the quickest and most efficient method of controlling the pandemic (1). A rapid cycle of research, development, and testing meant that vaccines became available for mass distribution barely 1 year after the novel virus was first identified.

The first confirmed case of COVID-19 in Saudi Arabia was in March 2020, and the first dose of the vaccine in Saudi Arabia was administered on December 17^th^ of 2020. As of December 2022, globally, there have been more than 645 million confirmed cases of COVID-19 and more than 6.6 million deaths (2). As of December 2022, over 69 million doses of the vaccine have been given to residents of Saudi Arabia and the total number of deaths attributed to COVID-19 in Saudi Arabia is 9,471 (3).

Saudi Arabia is fortunate to have a very robust, modern healthcare system, with free healthcare being offered to all citizens and legislation requiring that all residents have healthcare insurance (4). The Saudi government benevolently announced early on in the pandemic, that all citizens and residents alike would have access to free healthcare in the event of being infected with COVID-19.

The Saudi culture is generally very family-oriented. The typical living situation is families living together, often in multi-generational households, with unmarried members often not moving out until marriage. The average size of a Saudi household is 6.4 family members and 4.1 for a non-Saudi household (5). With the average number of members living together in a household in Saudi Arabia being so much higher and therefore more people living in close proximity, the chance of catching a communicable disease, such as COVID-19 can be expected to be comparatively higher than, for example, the United Kingdom where the 2021 average household size was only 2.36 (6). It has been estimated that older individuals are at higher risk of death from COVID-19 (7), thankfully in this respect, Saudi Arabia has a comparatively smaller population of older inhabitants, with only an estimated 3.2% of the population being aged 65 years and above (8), compared to almost six times the proportion (18.9%) of the U.K. population belonging to the same age range (9).

The expedited administration of the COVID-19 vaccine was shown to be crucial in reducing both the COVID-19-associated healthcare burden and the number of related deaths. One study estimates that approximately 168,000 hospitalizations were prevented and 59,000 lives saved in Brazil, with a hypothetical additional 104,000 hospitalizations that could have been prevented and 48,000 lives saved if they had carried out an accelerated COVID-19 vaccine rollout (10). Although Brazil's population is approximately six times that of Saudi Arabia, they have very similar health scores (63 and 61% respectively) (11), therefore accelerated vaccine rollouts in Saudi Arabia could have also led to a considerable number of avoided hospitalizations and deaths.

The reluctance or refusal to receive a vaccination commonly referred to as “vaccine hesitancy,” is a global concern. It was soon realized that a high percentage of the population would need immunity to curb the rapid spread of COVID-19. Several studies have found high levels of hesitancy (12–14). One meta-analysis (including studies from Saudi Arabia) reported global COVID-19 vaccine hesitancy between female and male respondents of 38.9–40.0% respectively (14). Another study of 36,958 Arabs, found a very high hesitancy rate amongst Arabs of between 81 and 83%, amongst the 3,588 Saudi respondents vaccine hesitancy was reported to be 78.7% (15).

Motivation for receiving any type of vaccine is a multifactorial matter across different sociodemographic groups. Several studies have been conducted regarding the attitudes of Saudi citizens and residents toward the COVID-19 vaccine. Non-adoption of the COVID-19 vaccine has been linked to gender, age, nationality, marital status, educational level, socioeconomic factors, the perceived risk from COVID-19 and underlying health conditions (1, 15–30). However, the majority of these other studies were conducted before the vaccine is available, therefore our survey can show the population perspective when the vaccine was already a reality.

One proposed theory is that the motivational rationale for vaccination can be classified as to *who* people take it for, for example, whether the reasoning behind the decision is that they take it for themselves or others (31). We aimed to examine the timing of registration for the vaccine and the motivation for residents of Saudi Arabia to take the COVID-19 vaccine. By understanding what motivated individuals in Saudi Arabia to receive the vaccine we hope to assist local public health decision-makers with future vaccine campaigns should a similar situation arise.

## Materials and methods

This was a cross-sectional survey of people living in Saudi Arabia. The inclusion criteria included all persons living in Saudi Arabia at the time of the survey. Exclusion criteria included anyone younger than 14 years old. The study was approved by King Fahad Medical City Institutional Review Board (approval number 21–342). No personal identifying information was gathered from the participants.

### Data collection tool

As we did not find a questionnaire in the literature that fully suited our purpose, therefore we constructed our own, drawing inspiration from some of the questions on previous questionnaires in the literature. This questionnaire was initially written in English and was tested for face and content validity among the authors, healthcare professionals and laypeople. Some changes were made, for example, we added additional reasons for taking the vaccine and a question regarding the willingness of taking a booster dose was added. The revised format was forward-translated into Arabic by two native Arabic speakers who have expertise in this field. Minor differences between their translations were agreed upon with the help of a third native speaker. The translation was then back-translated into English by two native English speakers. Any differences between the translations were agreed upon with the help of a third native speaker. The questionnaire was then piloted to a group of 20 people, from within our social network and amongst colleagues, and the feedback received facilitated some changes which were mainly minor semantic changes, but also, for example, included the expansion of an answer regarding who the respondent had felt pressured them to take the vaccine and removal of a question about the respondent's living situation. To reduce the primacy effect, that a respondent might be more likely to choose the first answers, we randomized the order of the answers for the electronic version.

The questionnaire encompassed the following two sections:

1. Demographic and clinical characteristics; which included age, gender, nationality, educational level, occupation, if working whether there is contact with other people and how often, and medical history, specifically if there is a medical condition from a checklist of the following conditions deemed to be higher risk if they contracted COVID-19 according to the CDC at that time: cardiac disease (heart failure, coronary artery disease, or cardiomyopathy), pregnancy and recent pregnancy, cancer, chronic kidney disease, diabetes mellitus, cerebrovascular disease, obesity, chronic pulmonary disease (COPD, asthma) other lung diseases, pulmonary fibrosis, pulmonary hypertension, Down syndrome, Human Immunodeficiency Virus, sickle cell disease, solid organ or blood stem cell transplantation, cystic fibrosis, thalassemia, immune deficiencies, liver disease, hypertension, and venous thromboembolism (32). Also, history of previous exposure to COVID-19 infection was recorded.
2. COVID-19 vaccination history; which comprised of whether or not the vaccine had been taken, plans to take a booster shot (if not done so), the timing of registration (due to the understandable demand for the vaccination, in some instances, there were delays for lower-risk individuals to be vaccinated; therefore, we asked about the timing of registration rather than the first dose date), the main reason for taking the vaccine or not taking the vaccine as appropriate. The 13 possible answers to the main reason for taking the vaccine in our questionnaire, were grouped into four categories namely: self-protection (taking it to protect oneself), indirect vaccination (taking it because someone recommended it/mandated it), paternalistic (taking it to protect one's family and loved ones) and altruistic reasons (taking it to protect the wider population). There was a big difference in the total number of responses between the different groups of rationale.
  Moreover, Vaccine hesitancy was addressed in this survey with a registration question, with the option to choose “I have not registered / nor had the vaccine until now.”

### Data collection procedure

Over a period of 3 months between January and March of 2022, we used a dual-based approach to questionnaire distribution. Utilizing both a paper-based format and an electronic link to the questionnaire which was distributed to patients, and visitors attending the outpatient clinics and also staff in King Fahad Medical City. The questionnaire was intended to be a self-administered questionnaire, however, in a few instances, we found that the respondent asked the distributor to help them in completing the questionnaire, which they did. Concurrently, an electronic version of the same questionnaire *via* the messaging application “WhatsApp” was distributed by the authors to the general public, creating a snowball sampling distribution. In this way, we were able to have a sample of both medically compromised and healthy respondents. The paper-based questionnaires were collected shortly after completion and the electronic version utilized the Google Forms software. All procedures for data collection were treated with confidentiality. The questionnaire included a statement that by proceeding the respondent implicitly agreed for the data in their responses to be utilized for the research analysis and that no personally identifiable information would be taken.

### Sampling technique and statistical analysis

The sampling technique was a convenience sample, and the sample size was estimated to be a minimum of 385 responses based on the estimated population of Saudi Arabia using the Raosoft^®^ sample size calculator (33).

All categorical variables, such as gender, nationality, education, etc., are presented as numbers and percentages. Continuous variable only age was expressed as the mean ± SD. Nonparametric tests were used when data were skewed. The Kolmogorov–Smirnov test was used to check the assumption of a normal distribution. Chi-square/Fisher's exact test was used according to whether the cell expected frequency was smaller than 5, and it was applied to determine the significant association between categorical variables. ANOVA was performed to determine the mean significant difference between age and vaccine registration. A two-tailed *p* < 0.05 was considered statistically significant. All data were entered and analyzed using the statistical package SPSS 25 (SPSS Inc., Chicago, IL, USA).

## Results

There were 820 responses in total, and after removing invalid responses, there were 802 participants included in the final analysis.

The sample included 521 female respondents (65%). The majority of respondents were Saudi citizens 710 (88.5%)and 99 non-Saudi respondents from 17 different countries.

**Figure 1:**
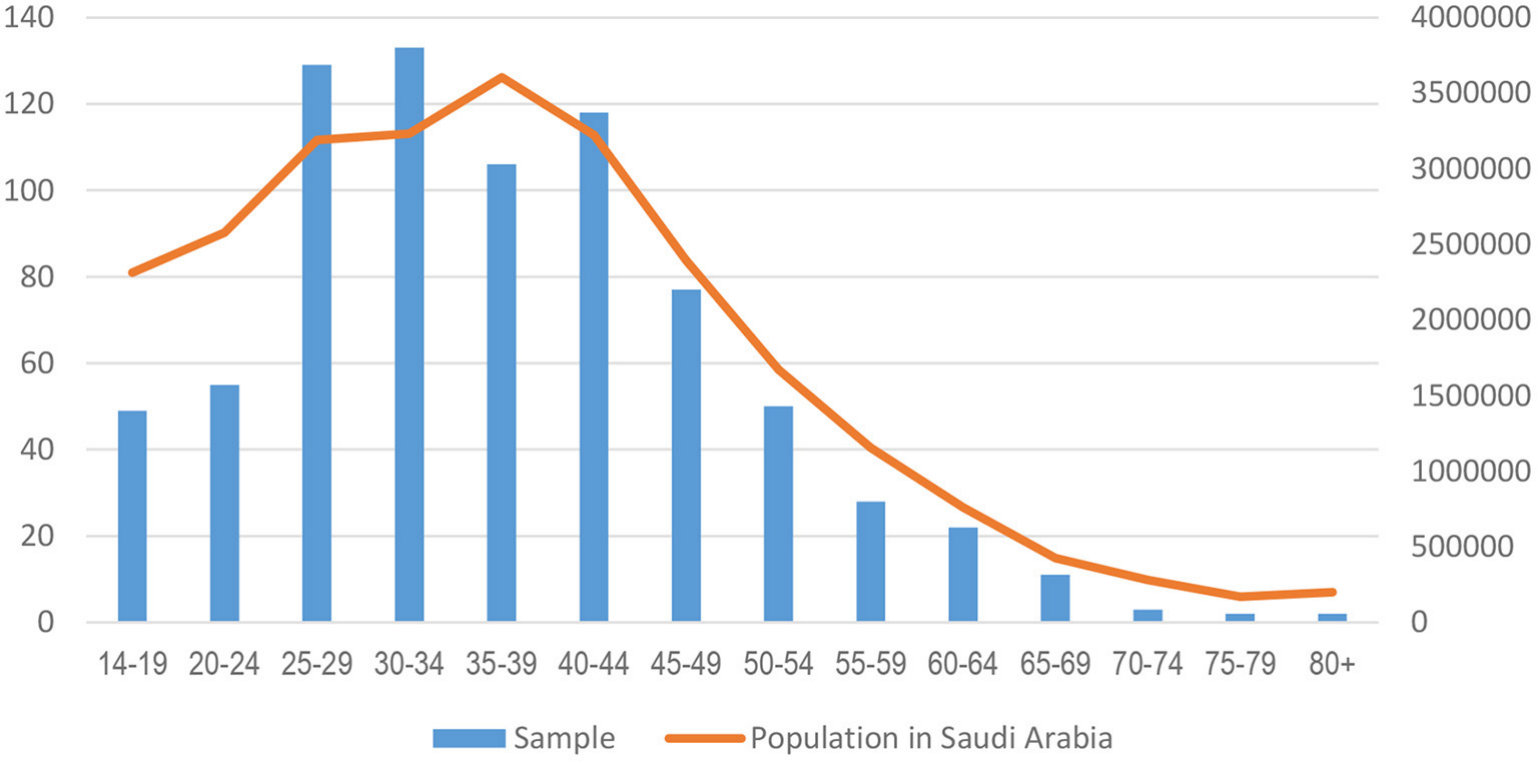
Age of respondents (in years) compared to the estimated population in Saudi Arabia.

The respondents were aged between 14 and 92, with a mean age of 36.96 ± 12.14. Figure 1 below shows that the sample follows the curvature of the Saudi population figures quite closely, especially from those aged 25 and above.

The majority of respondents (618 participants, 77.3%) stated that they had attained some college or higher level education, this is a lot higher than the general population due to the relatively high number of healthcare workers included in the sample, according to one estimate of the percentage of tertiary qualifications among Saudis aged between 25 and 64 calculates it to be approximately 30% (34).

Two hundred sixty-two (33.8%) respondents were healthcare workers. A large proportion of respondents, 225 (44.9%) stated that they worked in a job where there were multiple daily contacts with other people, and an additional 58 (11.6%) were working in an area specifically treating COVID-19 patients. 158 (31.5%) of the respondents were not working outside the home at the time of the survey; 60 (12.0%) respondents stated that they were working outside the home; however, they indicated that their contact with other people was “limited.”

The majority of respondents stated that they had not previously been infected with COVID-19 463 (58.9%), with 295 (37.5%) respondents stating that they had previously tested positive or suspected that they had been positive, and only 28 (3.6%) respondents were unsure whether they had previously had COVID-19 (Table 1).

**Table 1:** Demographic and clinical characteristics of the study participants (n = 802).

**Variables**	**Description**	***n* (n%)**
Gender	Male	281 (35.0%)
	Female	521 (65.0%)
Age (years)	Mean ± SD	36.96 ± 12.14
Nationality	Saudi	710 (88.5%)
	Non-Saudi	88 (10.9%)
Education	High school or below	181 (22.7%)
	College/university graduate/institution	518 (64.8%)
	Postgraduate	100 (12.5%)
Healthcare worker	Yes	262 (33.8%)
	No	514 (66.2%)
Occupation	At home	158 (31.5%)
	In a job where there are multiple daily contacts with people	225 (44.9%)
	In a job where there is limited contact with other people	60 (12.0%)
	In an area specifically treating COVID-19 positive patients	58 (11.6%)
Presence of medical condition	Yes	287 (35.8%)
	No	333 (41.5%)
	Unknown	182 (22.7%)
History of exposure to COVID-19	Close family members (with who I live with) were infected but I was not	102 (13.0%)
	I am not sure	28 (3.6%)
	I do not think I have ever had COVID-19	361 (45.9%)
	I think that I probably have had COVID-19 (but not proven through a test result)	39 (5.0%)
	I was previously infected with COVID-19 (as shown by a test result)	256 (32.6%)
Vaccination registration	As soon as available	496 (65.7%)
	I registered when I had to (because of regulations)	185 (24.5%)
	I registered when I noticed cases were increasing	74 (9.8%)
Reasons for taking the vaccine	Altruistic (e.g., to prevent the spread of COVID-19)	58 (7.5%)
	Indirect vaccination (e.g., government mandates)	240 (31.1%)
	Paternalistic (e.g., so as not to infect family members)	157 (20.4%)
	Self-protective (e.g., so as not to become infected)	316 (41.0%)

Categorical data are presented as frequencies (%), while continuous data are expressed as the mean ± SD.

Out of pre-listed medical conditions, the male participants accounted for 99 (34.3%) and the female participants for the remaining 188 (65.7%), which mirrors the distribution of males to females in the sample (35.0 and 65.0% respectively). The second major category of medical conditions for both male and female respondents was unknown (25.3 and 21.3% respectively), the respondents had not checked an option (Table 2).

**Table 2:** Study participants' medical conditions categorized by gender and age.

**Characteristics**	**Description**	**Females** ***n* = 521** **(% out of all female participants)**	**Males** ***n* = 281** **(% out of all male participants)**
Medical condition	No medical condition	211 (40.5%)	122 (43.4%)
	Unknown	111 (21.3%)	71 (25.3%)
	Anemia	43 (8.3%)	12 (4.3%)
	Diabetes	26 (5.0%)	21 (7.5%)
	Obesity	30 (5.8%)	15 (5.3%)
	Cancer	24 (4.6%)	9 (3.2%)
	Hypertension	17 (4.0%)	15 (5.3%)
	Cardiac	9 (3.3%)	13 (4.6%)
	Chronic pulmonary disease	12 (2.3%)	4 (1.4%)
	Venous thromboembolism (VTE)	11 (2.1%)	3 (1.1%)
	Immunocompromised	5 (0.96%)	1 (0.4%)
	Sickle cell disease	4 (0.8%)	2 (0.7%)
	Asthma	3 (0.6%)	0 (0.0%)
	Chronic kidney disease	0 (0%)	2 (0.7%)
	Downs syndrome	2 (0.4%)	0 (0.0%)
	Cystic fibrosis	1 (0.2%)	0 (0.0%)
	Liver disease	0 (0%)	1 (0.4%)
	Neurological	0 (0%)	1 (0.4%)
	Renal failure	1 (0.2%)	0 (0.0%)
		**Average age in years (females)**	**Average age in years (males)**
Medical condition	No medical condition	33.2	33.7
	Unknown	36.5	36.1
	Anemia	34.5	32.0
	Diabetes	46.3	50.8
	Obesity	40.1	38.3
	Cancer	45.4	42.5
	Hypertension	51.4	47.5
	Cardiac	38.3	59.3
	Chronic pulmonary disease	29.4	32.3
	Venous thromboembolism (VTE)	40.9	35.3
	Immunocompromised	26.8	45
	Sickle cell disease	33.0	20.5
	Asthma	28.0	^*^
	Chronic kidney disease	^*^	29
	Downs syndrome	14	^*^
	Cystic fibrosis	50	^*^
	Liver disease	^*^	75
	Neurological	^*^	53
	Renal failure	35	^*^

* No respondents with this medical condition.

**Figure 2:**
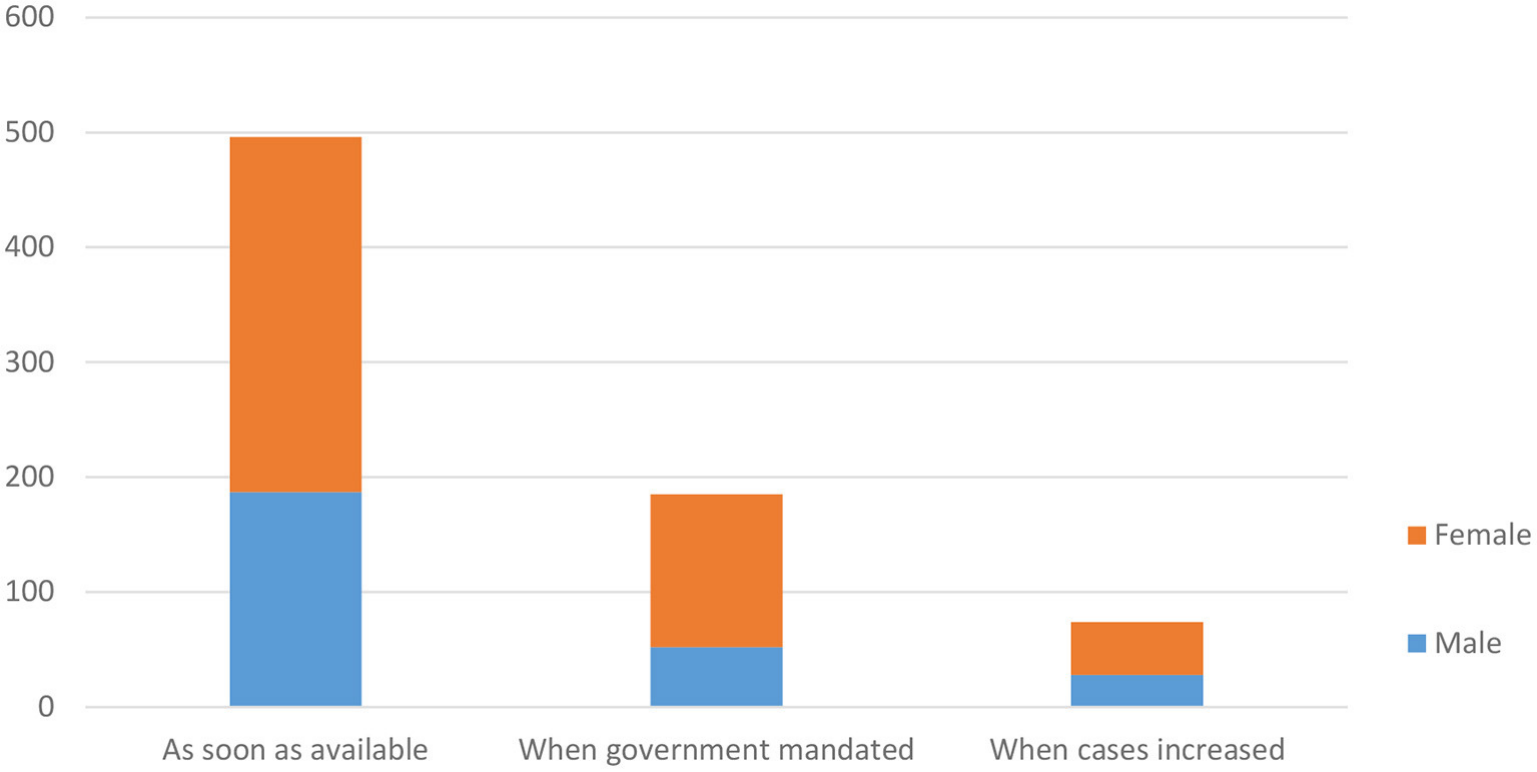
Timing of registration for the vaccine among the study participants.

Of those respondents who took the COVID-19 vaccine, the majority 65.7% of the participants either registered or went directly for vaccination as soon as the vaccine was available (Figure 2). The differences in gender and the timing of registration were not statistically significant (*P* = 0.057).

In our survey, only 11 (1.4%) of the respondents stated that they had not received the vaccine (at the time of the survey which was at least 1 year from the start of the vaccination program in Saudi Arabia). Of those who had not received the vaccine, they cited the following reasons: four cited medical conditions (including pregnancy and allergies), three cited accessibility of the vaccine as the main reason, and one said that they did not think that the COVID-19 disease would cause them a problem even if they caught it, and one expressed concern about the speed at which the vaccine had been produced. There was no reason stated for the remaining two.

**Figure 3:**
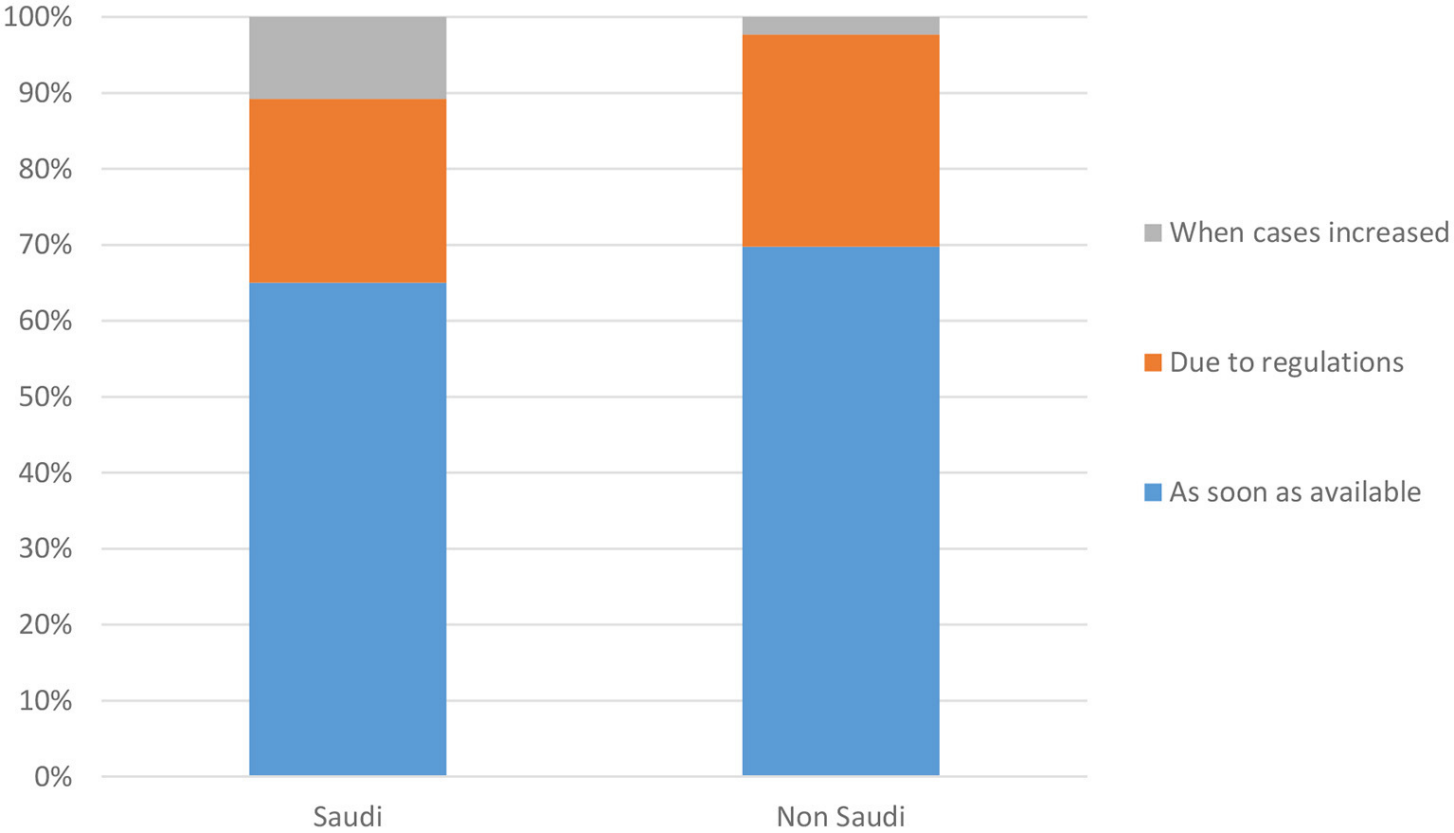
Nationality differences when respondents registered for the vaccine.

One notable difference between the Saudi and non-Saudi respondents was that more Saudi respondents indicated that they registered for the vaccine only when they noticed that the number of cases had started to increase. This finding was statistically significant (*P* = 0.044). The respondents were from 17 different countries, and the highest number of non-Saudi respondents were from the Philippines (30, 32.6% of the non-Saudis) (Figure 3).

**Figure 4:**
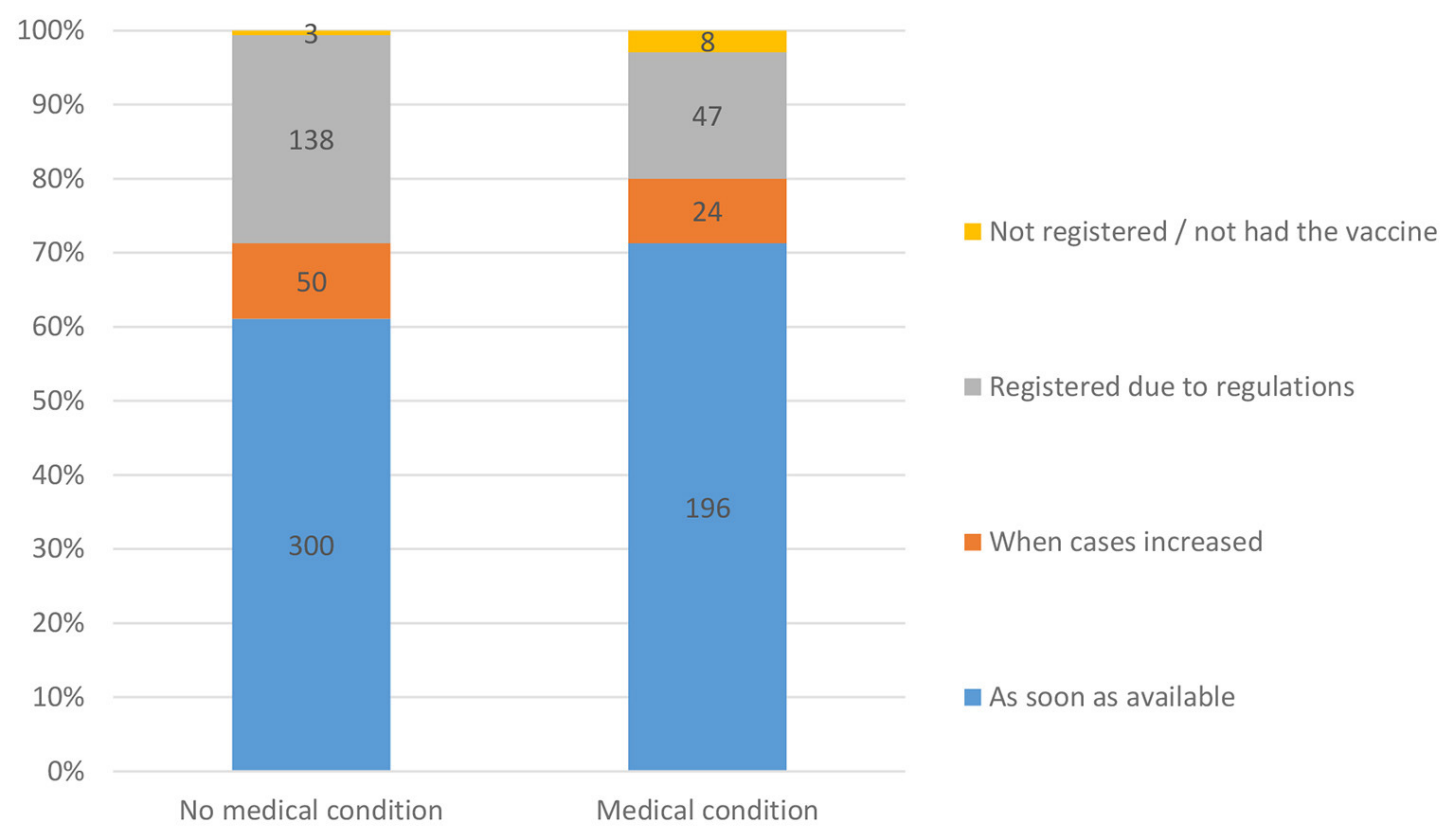
Respondents' willingness to register for vaccination as per the present or absence of medical condition.

A greater percentage of the participants with the medical conditions registered as soon as registration started (*P* = 0.022). Only 47 (25.4%) of those with medical conditions stated that the main reason was due to government mandated regulations, compared to 138 (74.6%) of those without medical conditions (Figure 4).

The respondent's previous history of COVID-19 infection was statistically significant only for those respondents who did not think that they had previously been infected with the virus, they were more likely to register as soon as the vaccine was available (*P* = 0.003) (Table 3).

**Table 3:** Relationship between vaccine registration and demographic and clinical characteristics.

**Variables**	**Description**	**Vaccination registration**			***P*-value**
		**As soon as available**	**I registered when I had to (because of regulations)**	**I registered when I noticed cases were increasing**	
Gender	Male	187 (37.7%)	52 (28.1%)	28 (37.8%)	0.057
	Female	309 (62.3%)	133 (71.9%)	46 (62.2%)	
Age (years)	Mean ± SD	37.28 ± 12.54	35.23 ± 10.12	36.00 ± 11.11	0.126
Nationality	Saudi	433 (87.8%)	161 (87.0%)	72 (97.3%)	^*^0.044
	Non-Saudi	60 (12.2%)	24 (13.0%)	2 (2.7%)	
Education	College/university graduate/institution	326 (66.1%)	122 (65.9%)	50 (67.6%)	0.998
	High school or below	110 (22.3%)	30 (16.2%)	19 (25.7%)	0.415
	Post graduate	57 (11.6%)	33 (17.8%)	5 (6.8%)	0.113
Healthcare worker	Yes	170 (35.5%)	65 (36.5%)	16 (21.9%)	0.057
	No	309 (64.5%)	113 (63.5%)	57 (78.1%)	
Occupation	At Home	93 (31.6%)	30 (22.4%)	19 (38.8%)	0.541
	In a job where there are multiple daily contacts with people	133 (45.2%)	66 (49.3%)	22 (44.9%)	0.275
	In a job where there is limited contact with other people	35 (11.9%)	18 (13.4%)	5 (10.2%)	0.834
	In an area specifically treating COVID-19 positive patients	33 (11.2%)	20 (14.9%)	3 (6.1%)	0.314
Presence of medical condition	Yes	194 (39.1%)	47 (25.4%)	24 (32.4%)	^*^0.022
	No	196 (39.5%)	86 (46.5%)	39 (52.7%)	0.187
	Unknown	106 (21.4%)	52 (28.1%)	11 (14.9%)	0.185
History of exposure to COVID-19	Close family members (with who I live with) were infected but I was not	67 (13.8%)	26 (14.2%)	4 (5.5%)	0.393
	I am not sure	15 (3.1%)	10 (5.5%)	3 (4.1%)	0.705
	I do not think I have ever had COVID-19	244 (50.1%)	60 (32.8%)	31 (42.5%)	^*^0.003
	I think that I probably have had COVID-19 (but not proven through a test result)	18 (3.7%)	15 (8.2%)	4 (5.5%)	0.211
	I was previously infected with COVID-19 (as shown by a test result)	143 (29.4%)	72 (39.3%)	31 (42.5%)	0.050
Reasons for taking the vaccine	Altruistic	46 (9.6%)	6 (3.3%)	5 (6.8%)	0.130
	Indirect vaccination	90 (18.7%)	119 (65.4%)	19 (25.7%)	^*^0.001
	Paternalistic	114 (23.7%)	18 (9.9%)	15 (20.3%)	^*^0.004
	Self-protective	231 (48.0%)	39 (21.4%)	35 (47.3%)	^*^0.001

Categorical data are presented as frequencies (%), while continuous data are expressed as the mean ± SD;

* shows that the P value is significant at P < 0.05.

The largest number of responses were categorized in the “self-protective” category 316 (41%). Only 30 (3.89%) responded that their medical condition was the main reason for them taking the vaccine. Those respondents in this category were more likely to take the vaccine as soon as it was available (*P* = 0.001).

The second largest category was for those whose responses were in the “indirect vaccination” category, with 240 (31.1%) responses. The majority of respondents said that they registered for the vaccine when mandated by regulations, this result was statistically significant *P* = 0.001. Eighteen (2.34%) of the respondents stated that travel was the main reason for taking the vaccine. Only 11.5% of the total respondents were non-Saudi, and almost two-thirds of those who responded that travel was the main reason for taking the vaccine were non-Saudi.

The third largest category was the “paternalistic” category, with 157 (20.4%) responses.

The smallest category was the “altruistic” category, with only 58 (7.5%) responses. These respondents answered that their main reason for taking the vaccination was to prevent the spread of COVID-19.

## Discussion

We found that the majority of respondents answered that the main reason for vaccination was of self-protection (Table 4). This echoes findings from another local study, where self-efficacy was the highest significant predictor of behavioral intentions toward COVID-19 (35). Given that the family unit is very important in Saudi Arabia, this finding was initially surprising for the authors, but after considering that Saudi Arabia is now considered only a slightly collectivist society, whereas previously it was considered a strongly collectivist society (36). Seen in this light it may be considered less surprising.

**Table 4:** Relationship between demographic/clinical characteristics and taking the vaccine.

**Variables**	**Description**	**Reasons for taking the vaccine**				***P*-value**
		**Altruistic**	**Indirect vaccination**	**Paternalistic**	**Self-protective**	
Gender	Male	19 (32.8%)	80 (33.5%)	70 (44.6%)	106 (33.5%)	0.079
	Female	39 (67.2%)	159 (66.5%)	87 (55.4%)	210 (66.5%)	
Nationality	Saudi	48 (82.8%)	212 (88.7%)	151 (97.4%)	271 (85.8%)	^*^0.001
	Non-Saudi	10 (17.2%)	27 (11.3%)	4 (2.6%)	45 (14.2%)	
Education	College/university graduate/institution	43 (74.1%)	145 (60.7%)	103 (65.6%)	210 (66.7%)	0.863
	High school or below	9 (15.5%)	56 (23.4%)	42 (26.8%)	63 (20.0%)	0.875
	Postgraduate	6 (10.3%)	38 (15.9%)	12 (7.6%)	42 (13.3%)	0.727
Healthcare worker	Yes	17 (29.8%)	72 (30.6%)	49 (33.8%)	117 (37.9%)	0.300
	No	40 (70.2%)	163 (69.4%)	96 (66.2%)	192 (62.1%)	
Occupation	At home	13 (32.5%)	43 (27.7%)	42 (42.9%)	52 (27.4%)	0.551
	In a job where there are multiple daily contacts with people	15 (37.5%)	67 (43.2%)	35 (35.7%)	101 (53.2%)	0.828
	In a job where there is limited contact with other people	9 (22.5%)	22 (14.2%)	11 (11.2%)	17 (8.9%)	0.508
	In an area specifically treating COVID-19 positive patients	3 (7.5%)	23 (14.8%)	10 (10.2%)	20 (10.5%)	0.968
Medical Condition	Yes	17 (29.3%)	77 (32.1%)	57 (36.3%)	123 (38.9%)	0.921
	No	26 (44.8%)	111 (46.3%)	71 (45.2%)	118 (37.3%)	0.796
	Unknown	15 (25.9%)	52 (21.7%)	29 (18.5%)	75 (23.7%)	0.988
History of exposure to COVID-19	Close family members (with who I live with) were infected but I was not	9 (15.8%)	20 (8.4%)	22 (14.6%)	47 (15.0%)	0.727
	I am not sure	1 (1.8%)	12 (5.1%)	5 (3.3%)	10 (3.2%)	0.988
	I do not think I have ever had COVID-19	24 (42.1%)	99 (41.8%)	80 (53.0%)	141 (45.0%)	0.918
	I think that I probably have had COVID-19 (but not proven through a test result)	2 (3.5%)	14 (5.9%)	2 (1.3%)	20 (6.4%)	0.690
	I was previously infected with COVID-19 (as shown by a test result)	21 (36.8%)	92 (38.8%)	42 (27.8%)	95 (30.4%)	0.605

Categorical data are presented as frequencies (%), while continuous data are expressed as the mean ± SD;

* shows that the P value is significant at P < 0.05.

The second largest group was those who were impacted by a decision made by someone other than themselves or someone who may “receive a primary benefit” from the vaccination, i.e., by government mandates. Saudi Arabia was fortunate in many respects that they were able to implement a very robust system of ensuring access to public areas was limited mainly to those who were not currently infected and either vaccinated or exempt from vaccination due to a medical condition. Because this group was so large, it cannot be denied that official mandates (regardless of their popularity), were effective in ensuring people were vaccinated. It is worth noting here that Saudi society is used to government mandates regarding vaccinations, there are existing regulations in place to ensure that other childhood vaccinations are completed before children entering full-time education, “anti-vaxxer” is not a term generally associated with Saudi Arabia.

**Figure 5:**
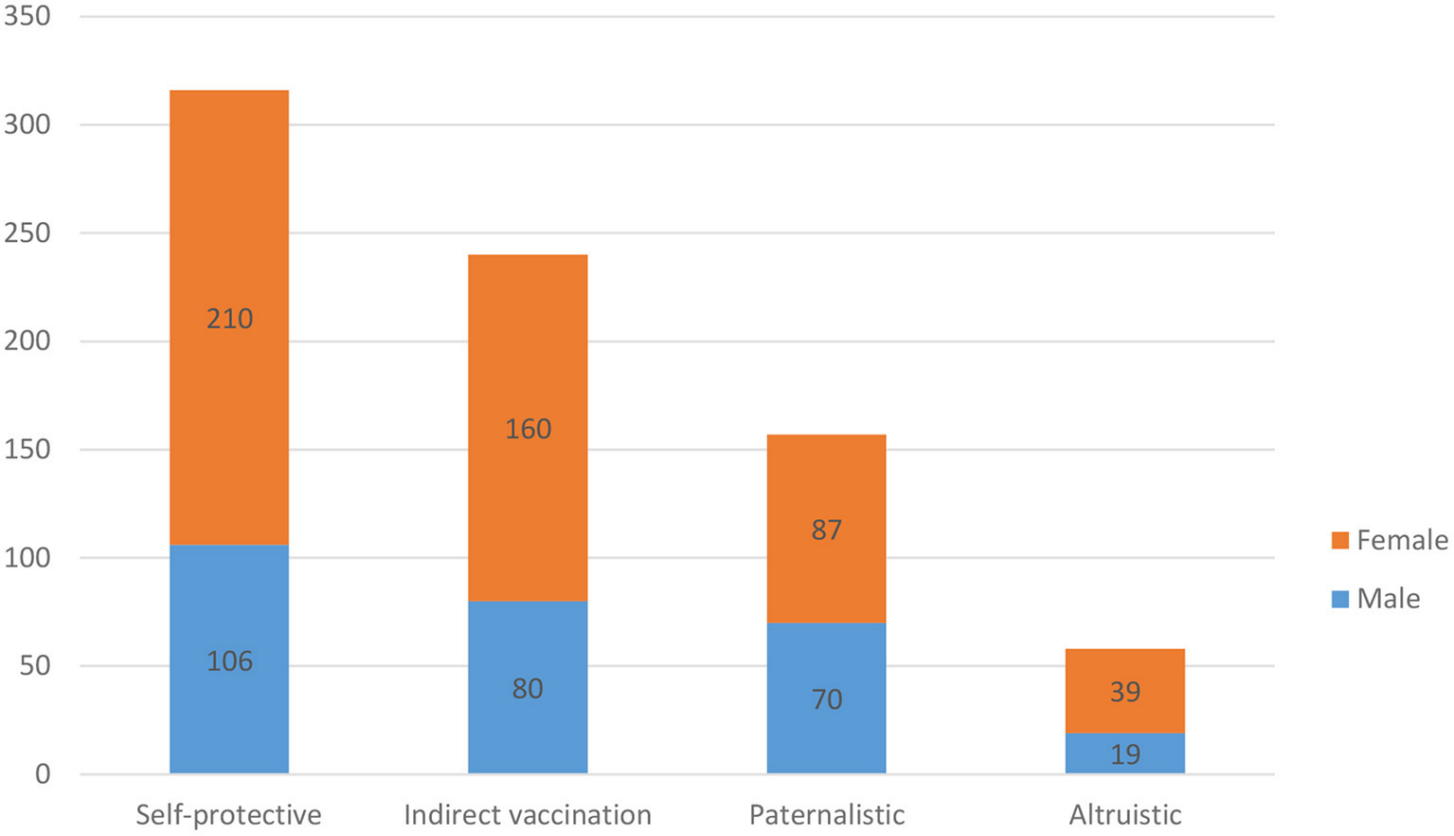
Respondents' reason for taking the vaccine according to the categorization of vaccine rationale and gender.

The third largest group of responses was that they took the vaccine for paternalistic reasons. A notable difference between the sexes was that comparatively more men answered with paternalistic types of reasons for being vaccinated. Saudi Arabia has a strong family structure and therefore it is not surprising that one-fifth of respondents (20.4%) said that their main reason for taking the vaccine was to protect family members. Part of the COVID-19 vaccination campaign in Saudi Arabia was aimed at evoking these paternalistic emotions, with billboard posters portraying elderly persons, with a phrase that the vaccination was there to protect them. However, when another study in a very different population is considered, the paternalistic rationale also is ranked high, being the third most common reason in a study of American workers (37). This result was statistically significant, with 75.7% of respondents noting that they had registered for the vaccine as soon as it was available (*P* = 0.004). This was the only category where there was a somewhat noticeable difference in gender percentages compared to the overall study sample. The male/female ratio for all respondents was 35% male and 65% female; however, the paternalistic rationale was cited by males in 44.6% of the responses compared to 55.4% for females (Figure 5).

Lastly, the smallest group of responses was in the altruistic category, the wish to protect others in general, i.e., outside of the immediate family group, accounting for only 7.5% of the responses. One behavioral study which considered altruism as a motivator compared to framed messages also found that people were generally less motivated by altruism (38).

The return to normalcy of the pre-pandemic times has been the goal of every country, some countries achieved this quicker than others and some are still implementing restrictions until now. Vaccination has been shown to be a major weapon in battling the spread of COVID-19. Speedy acceptance and implementation of vaccination programs have undoubtedly saved many lives and eased the tremendous economic burden which has been borne by governments around the world (10).

This survey has highlighted motivational factors for those taking the COVID-19 vaccine, by understanding the differences between the factors we can better allocate resources in future vaccine campaigns, should a similar situation arise. At the individual level, understanding that motivational factors have multifactorial backgrounds and therefore guidance could be individualized may help to guide healthcare workers in steering their patients toward vaccination.

The vast majority of respondents to the survey were Saudi (*n* = 710, 88.5%), and out of the non-Saudi respondents, 64.7% were healthcare workers (*n* = 57), which is not surprising considering the high percentage of non-Saudi healthcare workers working in Saudi Arabia (approximately 60%) (39). Nationality highlighted differences regarding when the respondents registered to receive the vaccine; non-Saudis were less likely to register due to noticing that the number of COVID-19 cases increased but were slightly more likely to register either due to regulations or to request vaccination as soon as it became available. This is because since August 1^st^, 2021, vaccination was mandated to enter the workplace (apart from those who were medically exempted) (40), and most adult non-Saudi residents are in Saudi Arabia on work visas.

Of the 265 respondents who indicated that they had one or more of the seventeen conditions listed and categorized by the CDC as being at higher risk from COVID-19 (see Table 2), only 30 (11.3%) stated that their medical condition was the most important reason for taking the vaccine. However, they were more likely to register for the vaccine as soon as it was available (194, 73.2%) compared to 196 (61.1%) with no medical conditions. A greater percentage of those with no medical conditions registered for the vaccine only when mandated 86 (26.8%) compared to 47 (17.7%) of those with medical conditions. These findings were statistically significant (*P* = 0.022). Although previous studies have indicated that people are concerned about the medical side effects of the vaccine (41), our sample appears to show that those with medical conditions were more likely to register early for vaccination, but only 17 (2.2%) of respondents indicated that the main reason they took the vaccine was upon advice from their physician. Physicians and other healthcare workers play a vital role in reducing patient apprehension about vaccination and if we are faced with a similar pandemic situation in the future, they should be prepared to pro-actively open a dialogue with patients about their intentions regarding the vaccine and answer any concerns that they might have regarding the effect of the vaccination in their particular medical situation.

The majority of respondents appeared to believe in the efficacy of the COVID-19 vaccine they answered that the main reason for receiving the vaccine was to prevent the occurrence or reduce the severity of infection, comparatively, very few respondents were convinced primarily by their physician (2.2%) however rather than this indicating the lack of trust in physicians it is probably because the majority of those respondents had medical conditions regarded to put them at higher risk. Although we don't have the figures for how many of the participants were advised to be vaccinated by their physician, but hadn't indicated that was the main reason for them to take the vaccine, we must not underestimate the value of physician advice to those patients, if a healthcare worker is themselves hesitant to be vaccinated this could be concerning for the public health authorities who may to some extent expect to rely on healthcare workers is a source of confidence and encouragement for the general public. One large study of healthcare workers found COVID-19 vaccine hesitancy levels ranging between 25.9 and 70.3% depending on race (42). One local study among women who were pregnant or planning to get pregnant indicated high levels of COVID-19 vaccine hesitancy (53.3–65.0%) (43).

Occupation did not appear to greatly impact the timing of vaccine registration. There was little difference between those in work environments with limited, multiple person-contact or even those in areas specifically treating COVID-19 patients; in fact, those mainly based at home were slightly more likely to register early on, and as they saw the number of cases increasing, they were less likely to register because of regulations. This lack of difference mirrors findings from another local study about preventive behaviors in healthcare workers between those being in the workforce and those who were not (35).

A previous history of infection with COVID-19 shows that a greater percentage of respondents who had not been infected or also had close family members who had been infected were more likely to register for the vaccine as soon as registration began compared to those who did not know whether they had previously had COVID-19. Unfortunately, within the context of this study, we do not know whether those who registered early were able to avoid COVID-19 because they were the precaution-taking type of people who also registered early or whether it was due to early registration and thus early immunity that helped them to thus far avoid infection.

In the case of future COVID-19 outbreaks or the emergence/re-emergence of other similar viruses, the government could utilize this information to better understand the reasons for Saudi citizens' and residents' willingness to take the vaccine to target those with vaccine hesitancy, although the importance of tailoring a campaign to an individual must not be underestimated.

Although not an objective of this study, previous studies have shown a correlation between COVID-19-related anxiety, trypanophobia and willingness to be vaccinated (44), the impact that this anxiety has on vaccine hesitancy specifically those amongst those living in Saudi Arabia could be studied in the future.

## Limitations

As the questionnaire was self-administered, we hoped to reduce any false reporting by the respondent being embarrassed by responding with a certain answer; conversely, there is always the risk that respondents may not understand the question correctly and therefore enter a wrong answer. However, this is an issue with any self-administered survey, we do not believe that the validity was impaired as from our experience with the questionnaires that we handed out, very few patients needed minor clarifications about the survey. Occasionally, some questions were left unanswered on the manually completed forms, and any questionnaires with the majority of questions unanswered were disregarded. We started the survey once we felt that a substantial number had received the vaccine, as the vaccine rollout was done in stages (higher risk first and ending with the lower risk, younger population) we had to wait for a few months; this delay may introduce some amount of recall bias. Due to the low numbers of those who had not taken the vaccine in our sample, we were unable to perform much analysis.

## Conclusions

The motivation to take the COVID-19 vaccination is multifactorial. By asking the question, for *who* do we take the vaccine, interesting insights appear. We found that most of our sample population appeared to take the vaccine with a view to self-protection, followed by indirect (for others) paternalistic and lastly altruistic reasons. Government regulations mandating vaccination appeared to have an effect in influencing a percentage of the population to register and become vaccinated, the percentage of vaccination among those living in Saudi Arabia is similar to countries without such widespread and enforceable mandates.

## Data availability statement

The raw data supporting the conclusions of this article will be made available by the authors, without undue reservation.

## Ethics statement

The studies involving human participants were reviewed and approved by King Fahad Medical City IRB. The patients/participants provided their written informed consent to participate in this study.

## Author contributions

All authors listed have made a substantial, direct, and intellectual contribution to the work and approved it for publication.
