# Supplementary material for: What motivated residents of Saudi Arabia to receive the COVID-19 vaccine?
Source: Front Public Health. 2023 Feb 7;11:1065157. doi: 10.3389/fpubh.2023.1065157 (PMC9941561; doi:10.3389/fpubh.2023.1065157)
Supplement: Supplementary file 1 [file Data_Sheet_1.docx]

**Supplemental material: Questionnaire**

We would be grateful if you could answer the following questions to help us understand the attitudes of people living in Saudi Arabia towards the Covid-19 vaccine. It should take approximately five minutes of your time. By continuing to complete this questionnaire you are agreeing to participate in this research, no personal identifying information will be collected from you. This research proposal was approved by the Institutional Review Board at King Fahad Medical City #21-342.

| 1.       Age __________  2.       Gender □ male □ female  3.       Nationality □ Saudi □ other (please state) _______________________________  4.       Educational level, please tick ONE:  o   High school or below  o   College / university graduate  o   Post graduate  5.       Occupation, tick the choice that best describes your current occupation:  o   Not healthcare worker   - Healthcare worker:   6. Occupation:   - In a job where there are multiple daily contacts with people   o In a job where there is limited contact with other people  o Working from home / not currently working / retired  o In an area specifically treating Covid-19 positive patients  7.       Do you have any of the following chronic diseases / medical conditions? (tick as many as apply to you now):  o   Cardiac disease (heart failure, coronary artery disease, or cardiomyopathy)  o   Cancer  o   Chronic kidney disease  o   Diabetes mellitus  o   Cerebrovascular disease  o   Obesity  o   Chronic pulmonary disease (COPD, asthma) other lung disease, pulmonary fibrosis, pulmonary hypertension  o   Down syndrome  o   HIV  o   Sickle cell disease  o   Solid organ or blood stem cell transplantation  o   Cystic fibrosis  o   Thalassemia  o   Immune deficiencies  o   Liver disease  o   Hypertension  o   Venous thromboembolism أمراض تخثر الدم  o   None of the above  8.       Your personal Covid-19 history is, choose ONE of the following:  o   I was previously infected with Covid-19 (as shown by a test result)  o   I suspect that I was previously infected with Covid-19 (but not proven through a test result)  o   Close family members were infected but I was not  o   I am not sure  o   I never had Covid-19  9.   Vaccination, please choose ONE of the following:  o   I registered for the vaccine as soon as registration began  o   I registered when I noticed cases were increasing  o   I registered when I had to (due to regulations)  o   I have not registered / nor had the vaccine until now  o   I went directly for the vaccine as soon as it was available (without registering)  10. Registration for Covid-19 vaccination started in December 2020. When did you first register for the vaccine (if you didn’t register for an appointment but went to receive the vaccine without an appointment use this date):  o   Date (if known)________________ / how many months ago? _________  11.   For those who have taken the vaccine please choose ONE (the MOST IMPORTANT reason) for you taking it:  o   By getting the COVID-19 vaccine, I believe that I will not infect family members  o   Getting the COVID-19 vaccine will prevent me from getting COVID-19 (make me immune)  o   I have medical conditions that put me at high risk of being sick if I get COVID-19  o   I am healthy but I believe that getting the COVID-19 vaccine will stop me being so sick in case I get COVID-19  o   Getting the COVID-19 vaccine will prevent spread of COVID-19  o   My physician advised me to get vaccinated  o   I have frequent contact with other people outside of my home (work, etc…)  o   It was authorized by my workplace  o   It was authorized by the government in order to enter certain places  o   My family pressured me to  o   Peer pressure  o   I saw other well-known people taking the vaccine  o   Travel (choose one):  o I cannot travel unless I am vaccinated  o I am worried about travelling if I am not vaccinated  o   Other _______________  12.   For those who HAVE NOT taken the vaccine please tick the ONE MOST IMPORTANT reason for not taking it for you personally:  o   Because I have medical conditions / allergies which prevent me  o   Because I am pregnant / breastfeeding  o   Because I don't feel that it is safe because the vaccine is still new and was tested too quickly  o   Because I am worried about the possible side-effects of the vaccine such as blood clots  o   Because I don't think that the vaccine is effective  o   Because I don't think COVID-19 is a real disease, it is a conspiracy  o   Because I don't think it will be a problem even if I get COVID-19  o   Because I have already had COVID-19, I think I am probably immune  o   Because it is inconvenient (time, location etc…)  o   Because the registration or appointment booking is difficult  o   Because I am careful enough, I don't think that I will contract COVID-19  o   Because I will wait for 'herd immunity' مناعة القطيع  o   Because I think that it will change my DNA  o   Other ___________________________________  14. If the Saudi Ministry of Health recommends a third dose of the vaccine, do you think that you will take it? |
| --- |
